# Antimicrobial Susceptibility Pattern of *Salmonella* spp. Isolated from Enteric Fever Patients in Nepal

**DOI:** 10.3390/idr13020037

**Published:** 2021-04-21

**Authors:** Anu Maharjan, Binod Dhungel, Anup Bastola, Upendra Thapa Shrestha, Nabaraj Adhikari, Megha Raj Banjara, Binod Lekhak, Prakash Ghimire, Komal Raj Rijal

**Affiliations:** 1 Central Department of Microbiology, Tribhuvan University, Kirtipur, Kathmandu 44618, Nepal; mhjanu14@gmail.com (A.M.); bwith.binod@gmail.com (B.D.); upendrats@gmail.com (U.T.S.); adhikarinaba13@gmail.com (N.A.); banjaramr@gmail.com (M.R.B.); binodlekhak9@gmail.com (B.L.); prakashghimire@gmail.com (P.G.); 2 Sukraraj Tropical and Infectious Disease Hospital, Teku, Kathmandu 44600, Nepal; docanup11@gmail.com

**Keywords:** enteric fever, *Salmonella enterica* Typhi, *S. enterica* Paratyphi A, blood culture, PMQR, MIC

## Abstract

Introduction: Enteric fever, a systemic infection caused by *Salmonella enterica* Typhi and *S. enterica* Paratyphi is one of the most common infections in developing countries such as Nepal. Aside from irrational practices of antibiotic use, mutations in chromosomal genes encoding DNA gyrase and Topoisomerase IV and by plasmid mediated quinolone resistant (PMQR) genes are suggested mechanisms for the development of resistance to nalidixic acid and reduced susceptibility to ciprofloxacin. Regardless of high endemicity of enteric fever in Nepal, there is paucity of studies on prevalence and drug-resistance of the pathogen. Therefore, this study aimed to assess the antibiotic susceptibility pattern of *Salmonella* isolates and determine the minimum inhibitory concentration of ciprofloxacin. Methods: A total of 1298 blood samples were obtained from patients with suspected enteric fever, attending Sukraraj Tropical and Infectious Disease Hospital (STIDH) during March–August, 2019. Blood samples were inoculated immediately into BACTEC culture bottles and further processed for isolation and identification of *Salmonella* Typhi and *S.* Paratyphi. Axenic cultures of the isolates were further subjected to antimicrobial susceptibility testing (AST) by using the modified Kirby–Bauer disc diffusion method based on the guidelines by CLSI. The minimum inhibitory concentration (MIC) of ciprofloxacin was determined by agar-dilution method. Results: Out of 1298 blood cultures, 40 (3.1%) were positive for *Salmonella* spp. among which 29 (72.5%) isolates were *S*. Typhi and 11 (27.5%) isolates were *S*. Paratyphi A. In AST, 12.5% (5/40), 15% (6/40) and 20% (8/40) of the *Salmonella* isolates were susceptible to nalidixic acid, ofloxacin and levofloxacin, respectively, whereas none of the isolates were susceptible to ciprofloxacin. The MIC value for ciprofloxacin ranged from 0.06-16 µg/mL in which, respectively, 5% (2/40) and 52.5% (21/40) of the isolates were susceptible and resistant to ciprofloxacin. None of the isolates showed multidrug-resistance (MDR) in this study. Conclusion: This study showed high prevalence of quinolone-resistant *Salmonella* spp., while there was marked re-emergence of susceptibilities to traditional first option drugs. Hence, conventional first-line-drugs and third-generation cephalosporins may find potential usage as the empirical drugs for enteric fever. Although our reporting was free of MDR strains, extensive surveillance, augmentation of diagnostic facilities and treatment protocol aided by AST report are recommended for addressing the escalating drug-resistance in the country.

## 1. Background

Enteric fever (typhoid and paratyphoid fever) is a serious bloodstream infection caused by *Salmonella* enteric serovar Typhi (*S.* Typhi) and Paratyphi (*S.* Paratyphi) A, B and C [1]. Enteric fever is transmitted predominantly by the fecal–oral route and manifests with several clinical outcomes including malaise, fever, chills, nausea, abdominal discomfort, transient rash, and hepatosplenomegaly [2]. In spite of increased sanitation, personal hygiene, and availability of effective treatment, enteric fever remains as a serious health problem in developing countries. An estimated 11.9–20.6 million cases of typhoid and paratyphoid fever with recorded mortality of 129,000–223,000 are reported annually from developing countries [3]. Moreover, a large proportion of these cases and mortalities is concentrated in South Asia, where it exhibits the seasonal variation, peaking in the rainy reason, from June to August [4]. In case of Nepal, the burden of enteric fever is concentrated in the densely populated regions: major city areas and low-lying southern Terai [5]. Reduced access to clean drinking water and proper sanitation facilities in these populous regions further deteriorates the quality of life, which leads to the frequent onset of infectious diseases including typhoid and paratyphoid fever. *Salmonella* has been estimated as the most common single pathogen isolated from blood cultures in Nepal, which is indicative of higher prevalence of enteric fever among Nepali population [6].

Antimicrobials such as chloramphenicol, ampicillin, co-trimoxazole and fluoroquinolones are the choice of drugs in the treatment of enteric fever and have proven to be the most effective agents [7]. The case fatality rate is estimated to be 10%–30% without antibiotic therapy, while the fatality rate falls to 1%–4% with the proper treatment course [2]. However, extensive and irrational prescription and use of these drugs have led to the emergence and spread of drug-resistance, often referred as multidrug-resistance (MDR) in the pathogenic strains of *Salmonella* [6]. MDR strains are responsible for treatment failures, narrowed option of drug regimens, and increased severities and mortalities [8]. The early emergence of drug-resistance among *Salmonella* isolates dates back to late 1980s when the traditional first-line drugs (chloramphenicol, ampicillin and trimethoprim-sulfamethoxazole) became ineffective due to antibiotic-resistance, which compelled clinicians to rely upon fluoroquinolones, especially ciprofloxacin [9]. Nonetheless, the recent global surge in resistance to fluoroquinolones could lead to a disastrous increase in global infectious diseases [10]. Aside from the inappropriate use of drugs, mutations in the quinolone resistance-determining region (QRDR) of DNA gyrase and topoisomerase IV are described as the reason for quinolones resistance. Subunits of the gyrase and topoisomerase IV are encoded, respectively, by *gyrA*, *gyrB*, *parC*, and *parE* genes. As the gyrA is the primary target of quinolones, alterations on these determinants help the pathogen to escape the action of antimicrobial agents [11,12]. Moreover, roles of other plasmid-mediated resistant determinants (PMQR) qnr genes and *aac (6*′*)-Ib-cr4* also have been described for the resistance to quinolones [13,14].

Nepal has endured several epidemics of enteric fever with varied patterns of drug resistance in the last few decades [15]. Since the very first report of MDR strain of *Salmonella* in 1991 [16], there has been continuous change in epidemiology and drug-susceptibilities. To cope with increasing resistance to first-line antibiotics, fluoroquinolones were introduced [17]. However, several findings suggest the increasing burden of nalidixic acid-resistant strains with reduced susceptibilities to quinolones, which subsequently has led to the introduction of third generation cephalosporin and azithromycin [18]. Consequently, these newer generations of drugs are also challenged by the MDR strains, as several previous reports have detected such drug-resistant isolates [16,19,20]. Several studies have suggested the re-emergence of antimicrobial susceptibilities of *Salmonella* spp. to the conventional first-line antibiotics, which can help fight the growing AMR in Nepal [15,17,18,21]. Adequate surveillance and effective infection controls serve as the pillars to curb the burgeoning AMR [22,23]. Despite several previous attempts, there is paucity of reliable data and ample scientific studies on the prevalence and antibiogram of *Salmonella* spp. This study was conducted to determine the prevalence and antibiotic susceptibility profile of *Salmonella* spp. isolated from clinical specimens obtained at a tertiary care hospital of Kathmandu, Nepal. This study further aimed to investigate if there was re-emergence of the susceptibility of the bacterial strains to the conventional drugs, as seen in some previous studies.

## 2. Material and Methods

### 2.1. Study Design and Sample Population

This cross-sectional study was conducted over a period of six months (March–August, 2019) at Sukraraj Tropical and Infectious Disease Hospital (STIDH), Teku, Kathmandu. The Sukraraj Tropical and Infectious Disease Hospital (STIDH) is the only Tropical Disease Hospital in Kathmandu. This is the referral hospital with availability of 100 inpatient beds. It receives referred patients from all over the country. A total of 1298 non-duplicated blood specimens were collected from the febrile-patients that were suspected to have enteric fever. Suspicion of enteric fever was based on one of the symptoms commonly reported in Nepal. Presence of one of these symptoms was used to suspect typhoid fever that included sustained fever that can be as high as 103 °F–104 °F; weakness; stomach pain; headache; diarrhea or constipation; cough; and loss of appetite. Since Nepal has a huge burden of typhoid illnesses, any person presenting with fever was suspected of typhoid fever unless it was clinically relatable to other conditions. Suspected patients of all age group and genders who provided written informed consent for their voluntary participation were included in the study. However, patients with incomplete demographic information and those with ongoing or prior antibiotic therapy were excluded from this study. A well-structured, pre-tested questionnaire was administered to each subject to record their demographic information, clinical history and prior antibiotic therapy. Only the recovered isolates of *Salmonella* were processed for further investigation. Duplication of isolates from the same patient was also avoided.

### 2.2. Sample Collection and Transport

Blood specimens were aseptically collected using vein puncture method by an experienced laboratory staff. The volume of blood sample was 8 mL–10 mL for adults and 2 mL–3 mL for pediatric patients. Collected blood samples were immediately transferred to BACTEC culture bottles containing brain heart infusion (BHI) broth (HiMedia, Bengaluru, Karnataka, India) [24].

### 2.3. Laboratory Processing and Identification of the Isolates

BACTEC culture bottles inoculated with blood specimens were incubated at 37 °C for up to 5 days. Isolates showing growth on BACTEC were further inoculated on conventional culture media such as blood agar (BA), Mac Conkey agar (MA) and xylose lysine deoxycholate agar (XLD). The inoculated culture plates were incubated at 37 °C for 18–24 h. The BA plates were used for the observation of non-hemolytic smooth white colonies, MA for nonlactose fermenting colonies and XLD for red colonies with black center [25]. Identification of the isolates was based on colony morphology, Gram staining, and biochemical tests including catalase test, oxidase test, methyl red (MR) test, Voges–Proskauer (VP) test, citrate utilization test, triple sugar iron (TSI) test, sulfide indole motility (SIM) test and urea hydrolysis test (urease test). Serotyping of the isolates was further performed by agglutination method using *Salmonella* polyvalent antisera O, monovalent O:2, O:9, O:12 and Vi for confirmation of different serovars [25].

### 2.4. Antimicrobial Susceptibility Testing (AST)

Antimicrobial susceptibilities of the Salmonella isolates were tested by using modified Kirby–Bauer disc diffusion in accordance with the guidelines outlined by the Clinical and Laboratory Standards Institute (CLSI) [26]. The antibiotic discs used were amoxicillin (AMX, 10 µg), chloramphenicol (C, 30 µg), cotrimoxazole (COT, 25 µg), nalidixic acid (NA, 30 µg), ciprofloxacin (CIP, 5 µg), ofloxacin (OF, 5 µg), levofloxacin (LEV, 5 µg), cefixime (CFM, 5 µg), cefotaxime (CTX, 30 µg), ceftriaxone (CTR, 30 µg) and azithromycin (AZM, 15 µg). In this method, broth culture of test organism (comparable to McFarland tube no. 0.5; inoculums density 1.5 × 10^8^ organisms/mL) was uniformly carpeted on the surface of MHA. Then, the antibiotic discs were placed over the lawn culture of the test organism, and the plates were incubated at 37 °C for 18 h (or overnight). After incubation, the diameter of zone of inhibition (ZOI) was measured and the results were interpreted as “Resistant” or “Intermediate” or “Susceptible” to that particular antibiotic based on the CLSI guidelines [26]. *Salmonella* isolates showing resistance to three or more than three antibiotics of different antibiotic classes were called MDR *Salmonella*. For instance, *Salmonella* spp. resistant to amoxicillin, chloramphenicol and cotrimoxazole were indicated as MDR *Salmonella* [27]. Control strains of *Escherichia coli* ATCC (American Type Culture Collection) 25955 and *Staphylococcus aureus* ATCC 25923 were used to ensure the standardization of susceptibility testing.

#### Determination of Minimum Inhibitory Concentrations (MICs)

Minimum inhibitory concentration of ciprofloxacin was determined by agar-dilution method [28] based on CLSI guidelines [26]. In this method, MHA plates with various concentration of ciprofloxacin (ranging from 0.0625 µg/mL to 32 µg/mL) were prepared and the test organisms were inoculated on the agar surface. After proper inoculation, the plates were incubated at 37 °C for 18–20 h. Following sufficient incubation, the results were interpreted as “sensitive” or “resistant” using breakpoints approved by CLSI [26].

### 2.5. Data Analysis

Data collected through the laboratory analyses were entered in Microsoft Excel 2016. Data were analyzed by using statistical package for social science (SPSS) version 24.0. Chi-squared (χ^2^) test was used to predict the relationship between the variables in which a *p* value of <0.05 was considered as statistically significant.

## 3. Results

**Figure 1:**
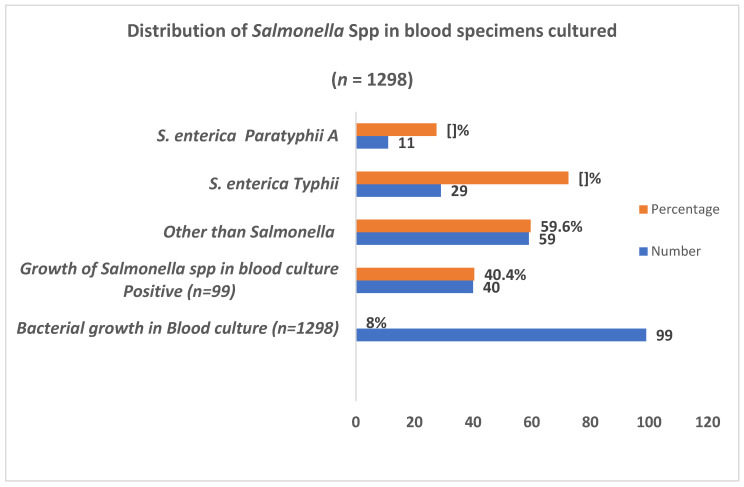
Distribution of *Salmonella* spp. in blood specimens cultured.

Among the total specimens processed, 7.6% (99/1298) showed visible growth of different microorganisms whereas 3.1% (40/1298) specimens were positive for the isolation of *Salmonella* spp. Of the total *Salmonella* isolates 72.5% (29/40) were *S. enterica* Typhi and 11 27.5% (11/40) were *S. enterica* Paratyphi A (Figure 1).

### 3.1. Distribution of Salmonella Positive Cases by Months

**Figure 2:**
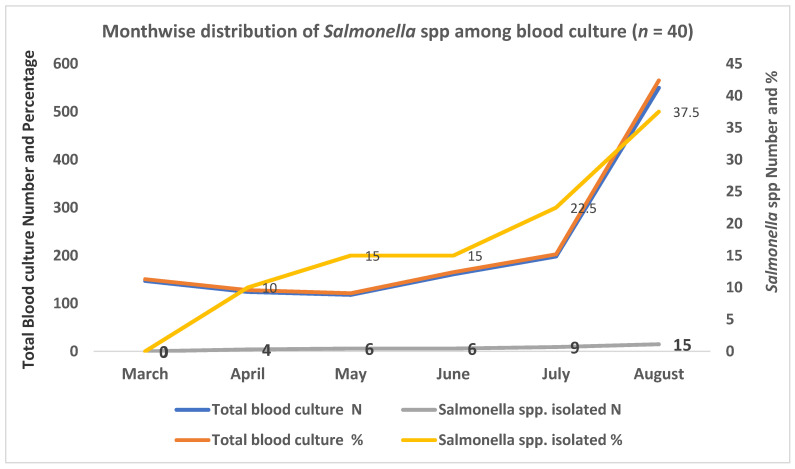
Monthwise isolation of *Salmonella* spp. from blood specimens.

During the six months of the study, the highest number (15/40) of *Salmonella* spp. were isolated in August and the lowest (4/40) in April whereas none of the *Salmonella* spp. were isolated in the month of March (Figure 2).

### 3.2. Sample Distribution According to the Gender and Age Group

Of the total samples collected, 59.5% (772/1298) and 40.5% (526/1298) were obtained from male and female subjects, respectively. Similarly, the growth positivity rates of the sample were 3.4% (26/772) and 2.7% (14/526) for male and female patients, respectively. Among 40 isolates, 65% were obtained from male and 35% were from female patients. There was a significant association of the burden of cases and isolates with the gender from which samples were collected. In the age-group distribution, 30.1% (391/1298) of the samples were obtained from patients of age group 21–30 years old, followed by 18.3% (285/1298) of the cases from the age group of 11–20. Similarly, the highest number (19/40; 47.5%) of isolates was obtained from the age group of 21–30 followed by the age group of 11–20 years old (13/40; 32.5%) (Table 1).

### 3.3. Antibiotic Susceptibility Pattern of Salmonella Isolates

The highest rate of antibiotic susceptibility was observed towards cefixime (100%) followed by azithromycin (97.5%) and ceftriaxone (95%), whereas the lowest rate of susceptibility was observed towards nalidixic acid (12.5%), followed by ofloxacin (15%) and levofloxacin (20%). All of the isolates were susceptible to all classes of antibiotics used, such as amoxicillin, chloramphenicol, and cotrimoxazole. Therefore, none of the isolates were reported as multidrug resistant (MDR) (Table 2).

### 3.4. Nalidixic Acid Susceptibility Pattern of the Isolates

**Figure 3:**
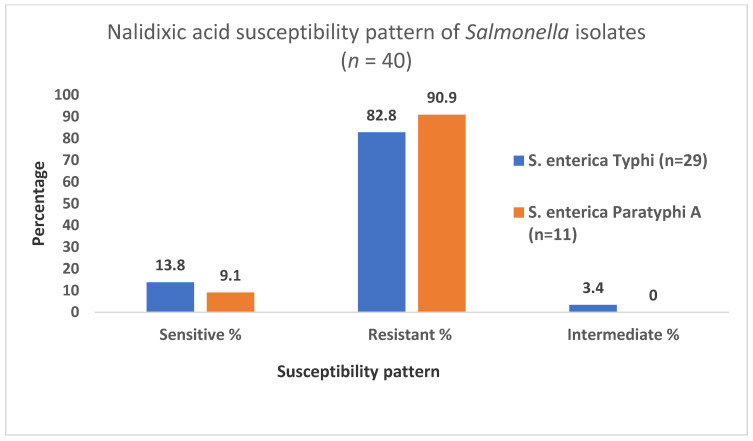
Nalidixic acid susceptibility pattern of *S. enterica* Typhi (*n* = 29) and *S. enterica* Paratyphi A (*n* = 11).

Out of 29 *S. enterica* Typhi, 4 (13.8%) isolates were susceptible towards nalidixic acid and 24 (82.8%) were resistant. Similarly, out of 11 *S. enterica* Paratyphi A, 1 (9.1%) isolate was susceptible towards nalidixic acid while 10 (90.9%) of the isolates were resistant to it. Although the antibiotic susceptibility rate was not significantly associated with the serovar, *S. enterica* Paratyphi A strains showed a higher rate (90.9%) of resistance to nalidixic acid than that of *S. enterica* Typhi (82.8%) (Figure 3).

### 3.5. Determination of Minimum Inhibitory Concentrations (MIC) of Ciprofloxacin

In this study, the MIC values for ciprofloxacin ranged from 0.06 µg/mL–16 µg/mL. The test detected 5.0% (2/40) isolates with MIC of ≤0.06 µg/mL as susceptible strains. On the contrary, 52.5% (21/40) isolates had MIC ≥ 1 µg/mL and were labeled as resistant, whereas 42.5% (17/40) isolates had MIC between 0.12 µg/mL–0.5 µg/mL, showing decreased susceptibility to ciprofloxacin (Table 3).

In the disc diffusion method, none of the isolates were susceptible, 65% (26/40) isolates were intermediate and 35% (14/40) isolates were resistant to ciprofloxacin in the susceptibility assay by disc diffusion method (Table 4).

In the MIC determination assay, the distribution of the isolates according to their serovars was also assessed. Out of 29 isolates of *S. enterica* Typhi, 2 (6.9%) were susceptible, 11 (37.9%) were resistant and 16 (55.2%) showed reduced susceptibility to ciprofloxacin. Similarly, out of total (11) *S. enterica* Paratyphi A, 10 isolates (99.9%) were resistant and 1 (9.1%) isolate showed reduced susceptibility to ciprofloxacin (Table 5).

## 4. Discussion

Enteric fever is one of the major endemic diseases of low-to-middle-income countries (LMICs) like Nepal. The presence of densely populated urban areas with reduced access to safe drinking water and sanitation, lowered socio-economic status, lack of effective surveillance and poor infection control are some of the driving factors for the high endemicity of the diseases in these countries [29]. Due to the lack of extensive surveillance on the prevalence of enteric fever and unavailability of reliable data on antimicrobial resistance, Nepal is facing several challenges relating to controlling the disease burden and growing drug-resistance [6]. This study was conducted to fulfill a research gap in the country, thereby attempting to estimate the prevalence of enteric fever and the drug-resistance of the pathogens isolated from suspected patients visiting a tertiary care hospital of Kathmandu city over a period of six months. In addition, we also tried to predict whether there was re-emergence of the susceptibility of enteric pathogens to the conventional first-line drugs. In this study, significant prevalence of enteric fever and drug-resistance profile of *Salmonella* was observed, which suggests the urgency of intervention measures to counteract the AMR [10]. At the same time, the effectiveness of the first-line antibiotics was well observed, which is suggestive of the possibility of the re-introduction of those antibiotics into the treatment regimens.

In this study, less than one-tenth (7.7%) of the total specimens was positive for bacterial growth, among which 3.1% of the specimens was positive for the growth for *S. enterica* (*S. enterica* Typhi and *S. enterica* Paratyphi A). This finding was in accordance with the previous various studies reported from Kanti children’s Hospital (2%), Kathmandu [30], and International Children Friendship Hospital (4.9%), Kathmandu, Nepal [15]. The low growth rate in blood culture can be attributable to the practices of self-medication, which is more common in developing countries [31] including Nepal [32], although we excluded the subjects who had undergone antibiotic therapy for past three months. In this study, the prevalence of *S. enterica* Typhi (72.5%) was much higher in comparison to *S. enterica* Paratyphi A (27.5%). This finding is similar to the previous studies conducted in Nepal [15,18,33,34]. Although there is no such well-established reason for serotypic variation in enteric fever, the higher incidence of *S. enterica* Typhi might be due to the waterborne transmission of *S. enterica* Typhi, as it usually involves smaller inocula than *S. enterica* Paratyphi, with the latter being achieved through the food-borne transmission that requires larger inocula [35].

In our study the higher incidence of the typhoid and paratyphoidal fever was observed in the month of August. Enteric fever cases occur sporadically throughout the year, peaking in the summer and the rainy season [32,35]. During this season, floods and seepage of water treatment plants and/or sewers can contaminate drinking water and food, thereby resulting in the higher number of typhoid and paratyphoidal cases.

Although there was not any association of gender with the incidence of disease, our study showed a higher incidence of cases in males (65%) in comparison to females (35%). A slightly different male to female ratio was reported by some previous studies [36,37]. The preponderance of enteric fever among the male population may be explained by their involvement in outdoor activities more frequently, exposing them to the source of infection.

In this study, patients of the age group 21–30 years (47.5%) were the most affected by enteric fever, followed by the age group 11–20 years (32.5%). This is in accordance with the findings of the previous studies reported in Kathmandu valley [35,38]. The higher concentration of disease burden among younger adults may be attributable to their active social life where the habit of eating out is very likely. In the present study, all the *Salmonella* isolates were susceptible to chloramphenicol, amoxicillin and cotrimoxazole, implying that none of the isolates was multidrug-resistant. This finding was similar to study reported from Alka hospital, Lalitpur [18]. In another study conducted in China and Indonesia, no MDR serovar of *S. enterica* Typhi were identified from China and Indonesia [39]. In another study, respectively, 98.2% and all of the isolates of *S. enterica* Typhi were susceptible to ampicillin and chloramphenicol. In the same study, all of the *S. enterica* Para Typhi A isolates were susceptible to ampicillin and cotrimoxazole, whereas 96.7% of those isolates were susceptible to chloramphenicol [18]. However, the present finding was in harmony with the study conducted by Joshi et al., 2018 [38] and contrary to the study conducted by Shrestha and Basnet, 2019 [40]. The first incidence of MDR among *Salmonella* serovars (*S. enterica* Typhi and *S. enterica* Paratyphi) was reported during the late 1990s and was believed due to the presence of R plasmid [41,42,43]. The emergence of MDR strains led to the switching of treatment options from conventional first-line drugs to the newer generation (or second-line) drug such as fluoroquinolones [44]. Thus, early discontinuation of chloramphenicol, ampicillin and cotrimoxazole in treatment options since the vey onset of MDR strains may be the reason for the re-emergence of strains susceptible to first-line therapeutic options. In addition, the absence or loss of the plasmid, often due to mutations responsible for resistance to these first-line drugs may also be the reason for the revival of the susceptibility of the strains [18].

In this study, all the isolates showed susceptibility towards cefixime, which is similar to the earlier findings [17,38,45]. However, these studies also reported the absence of resistant strains (all of the isolates were susceptible) towards cefotaxime and ceftriaxone. In our study, respectively, more than two-thirds (72.5%) and the 5% of the isolates were susceptible and resistant to cefotaxime, whereas 97.5% of the isolates were susceptible and to ceftriaxone. Third generation cephalosporins have been used to treat MDR and fluoroquinolone resistant strains [18,46] but resistance to extended-spectrum cephalosporins by production of ESBL enzymes have been reported in many countries [47,48,49,50]. In South Asia, cephalosporins such as ceftriaxone and cefixime are currently the mainstay of treatment for enteric fever, and are often started empirically, possibly driving resistance in typhoid [51]. This study showed 97.5% susceptibility towards azithromycin, which is similar to the previous findings [38,45]. Azithromycin has been found effective against uncomplicated enteric fever but there are certain sporadic reports of azithromycin resistance as reported by some previous studies from India [52,53].

The study showed 12.5%, 15% and 20% sensitivity to nalidixic acid, ofloxacin and levofloxacin, respectively. A high proportion (85%) of the isolates showed reduced susceptibility to nalidixic acid, while more than one-third (35%) showed reduced susceptibility to ciprofloxacin. Although there was no significant association of serotype with antibiotic susceptibility rate, *S. enterica* Paratyphi A strains showed higher rate of nalidixic acid resistance than *S. enterica* Typhi. None of the isolates were susceptible to ciprofloxacin in disc diffusion test while two of the isolates were found susceptible according to MIC breakpoint having MIC value ≤ 0.0625 µg/mL. A report from the previous study conducted in the period of 2002–2014 was consistent with our findings [24]. Resistance to nalidixic acid is associated with decrease in fluoroquinolone susceptibility and an inevitable evolution of fluoroquinolone resistance [31]. There is gradual increase in ciprofloxacin non-susceptibility implied by various studies, ranging from 5% in *S. enterica* Typhi and 13% in *S. enterica* Paratyphi A during 1993–2003 [33], 0% in *S. enterica* Typhi and 3.3% *S. enterica* Paratyphi A resistance in 2011–2012 [18] and 93.7% non-susceptibility (both resistant and intermediate) during 2012–2016 [38].

MIC of ciprofloxacin for *S. enterica* Typhi and *S. enterica* Paratyphi A ranged from 0.06 µg/mL–16 µg/mL. In a previous study reported from Kathmandu Valley, the MIC value ranged within 0.125–8 µg/mL [34]. An increasing trend of ciprofloxacin resistance was observed with *S. enterica* Typhi from 29.6%–37.2% during 2010–2012 in which overall resistance was higher in *S. enterica* Paratyphi A (49.3%) compared to that of *S. enterica* Typhi (34.5%) [53]. A study by Geetha et al. (2014) has reported strains of typhoidal salmonellae showing MIC value of ≥1 µg/mL for the detection of PMQR determinants and has reported prevalence of PMQR determinants for the first time in India [54]. PMQR genes facilitates selection of higher level of resistance against quinolones though they do not reach CLSI breakpoint [55]. Plasmid-mediated mechanisms typically confer reduced susceptibility to ciprofloxacin in the MIC range of 0.125 µg/mL to 1.0 µg/mL and a modest increase in susceptibility to nalidixic acid in the MIC range of 8 µg/mL to 32 µg/mL [56,57,58].

Fluroquinolones, especially nalidixic acid are the choice of drugs against enteric fever in LMICs due to their cost-efficiency, accessibility and availability in oral pills and tablets. However, their long-held utility is now challenged by the burgeoning strains of nalidixic-acid-resistant *Salmonella* (NARS). Genetic factors such as mutations in the genes coding for DNA gyrase (gyrA and gyrB) and topoisomersase IV (parC and pare) are a suggested mechanism for emergence of nalidixic-resistance [59]. Therefore, the role of genetic factors can also be attributable to the high prevalence of NARS in our study; however, dealing with genetic factors was beyond the scope of our study design. As seen in several previous studies [15,18,21], we also observed a high prevalence of quinolones and nalidixic-acid resistant strains while there were notably higher susceptibility rates towards conventional first-line drugs (chloramphenicol, ampicillin and trimethoprim-sulfamethoxazole) and third generation cephalosporins (ceftriaxone, cefotaxime, and cefixime). This sort of rare reemergence of susceptibility could be due to the obsolete status of conventional antibiotics for a long time. In addition to this, due to the loss of high molecular weight self-transmissible resistant-inducing plasmids in the pathogenic strains in the course of evolution and mutation [17,21].

A choice of single antibiotic is no longer an effective option in dealing with increasing AMR among *Salmonella* isolates. As suggested by previous studies, a combination of third-generation cephalosporins and azithromycin can better compensate than when used alone [60]. Our findings also support the effectiveness of these classes of antibiotics, as seen in several previous studies [17,21].

### Limitations of the Study

This study was conducted in a single hospital for a time period of six months, covering a small population from a limited geographical region. Therefore, the findings of our study may not be representative of the extended geographical regions of the country. Furthermore, we could not establish the role of several socioeconomic factors such as sanitation and hygiene, over-the-counter (OTC) use of drugs, and knowledge, attitude and practice (KAP) of antibiotic prescription and use among the healthcare workers and the patients on the prevalence of the burden of disease. This study did not establish the role of genetic factors in conferring the drug-resistance to the bacterial strains. Therefore, further molecular study consisting of green synthesized nanomaterials in the optical biosensor devices is suggested in future studies to explain the possible mechanisms [61,62].

## 5. Conclusions

This study revealed a high rate of resistance among *Salmonella* isolates to fluoroquinolones, whereas the re-emergence of susceptibility was observed to the conventional drugs including third-generation cephalosporins, which is suggestive of possible re-introduction of such drugs in case management. Our study reported the absence of MDR strains. On the diagnostic side, the detection of drug-resistance by the determination of the MIC can offer better value than using disk-diffusion alone. Proper infection control, the provision of diagnostic facilities, and adherence to the rational practice of antibiotic prescription and use are the recommended measures to cope with the heightened endemicity of enteric fever in the country.

## Figures and Tables

**Table 1:** Distribution of *Salmonella* isolates according to age and gender of patients.

Character	Total Blood Culture Subjected	*Salmonella* spp. Isolated	
Gender	*n* (%)	*n* (%)	*p*-Value
Male	772 (59.5)	26 (65)	0.4
Female	526 (40.5)	14(35)	
Total	1298	40	
Age group in (years)			
0–10	43 (3.3)	2 (5)	0.002
11–20	238 (18.3)	13 (32.5)	
21–30	391 (30.1)	19 (47.5)	
31–40	267 (20.6)	5 (12.5)	
41–50	159 (12.3)	1 (2.5)	
>51	200 (15.4)	0	

**Table 2:** Antibiotic susceptibility pattern of *Salmonella* isolates.

Antibiotics	Total Isolates	Antibiotic Susceptibility Pattern of *Salmonella* Isolates					
		Sensitive		Resistant		Intermediate	
		*n*	%	*n*	%	*n*	%
Amoxicillin	40	40	100	0	0.0	0	0.0
Chloramphenicol		40	100	0	0.0	0	0.0
Cotrimoxazole		40	100	0	0.0	0	0.0
Nalidixic acid		5	12.5	34	85.0	1	2.5
Ciprofloxacin		0	0.0	14	35.0	26	65.0
Ofloxacin		6	15.0	1	2.5	33	82.5
Levofloxacin		8	20.0	1	2.5	31	77.5
Cefixime		40	100	0	0.0	0	0.0
Cefotaxime		29	72.5	2	5.0	9	22.5
Ceftriaxone		38	95.0	1	2.5	1	2.5
Azithromycin		39	97.5	1	2.5	0	0.0

**Table 3:** Minimum inhibitory concentration of ciprofloxacin against *Salmonella* isolates.

Organism	Total *n* (%)	MIC Breakpoint of Ciprofloxacin *n* (%)		
		Sensitive ≤0.0625	Intermediate 0.125–0.5	Resistant ≥1
*Salmonella* spp.	40 (100)	2 (5.0%)	17 (42.5%)	21 (52.5%)

**Table 4:** Comparison of susceptibility pattern to ciprofloxacin disc diffusion method and ciprofloxacin MIC.

Test Performed	Total *n* (%)	Sensitivity Pattern of Ciprofloxacin *n* (%)			
		Sensitive	Resistant	Intermediate	*p*-Value
Disc diffusion method	40 (100)	0	14 (35)	26 (65)	0.07
MIC	40 (100)	2 (5)	21 (52.5)	17 (42.5)	

**Table 5:** MIC of ciprofloxacin and nalidixic acid screening test for *S. enterica* Typhi and *S. enterica* Paratyphi A.

MIC (µg/mL)	*S. enterica* Typhi (*n* = 29)			Sensitivity Pattern for Ciprofloxacin	*S. enterica* Paratyphi A (*n* = 11)			Sensitivity Pattern for Ciprofloxacin
	NAS	NAI	NAR		NAS	NAI	NAR	
0.06	2			Sensitive (*n* = 2) 6.9%				
0.12				Intermediate (*n* = 16) 55.2%	1			Intermediate (*n* = 1) 9.1%
0.25	2							
0.5		1	13					
1			7	Resistant (*n* = 11) 37.9%			7	Resistant (*n* = 10) 99.9%
2			2				3	
4								
8			1					
16			1					
Total	4	1	24		1		10	

## Abbreviations

AMR: Antimicrobial resistance; AST: Antimicrobial susceptibility test; ATCC: American Type Culture Collection; BA: Blood Agar; BHI: Brain Heart Infusion; CFU: Colony Forming Unit; CLSI: Clinical Laboratory and Standard Institute; DNA: Deoxyribose Nucleic Acid; DoHS: Department of Health Services; ENPHO: Environment and Public Health Organization; ESBL: Extended Spectrum Beta-Lactamase; EUCAST: European Committee for Antimicrobial Susceptibility Testing; IncHI: Incompatibility Type HI; LB: Luria Bertani; MA: Mac- Conkey Agar; MDR: Multidrug Resistant; MHA: Muller Hinton Agar; MIC: Minimum Inhibitory Concentration; MR: Methyl Red; NAI: Nalidixic Acid Intermediate; NAR: Nalidixic Acid Resistant; NAS: Nalidixic Acid Sensitive; NB: Nutrient Broth; PCR: Polymerase Chain Reaction; PMQR: Plasmid Mediated Quinolone Resistance; QRDR: Quinolone Resistant Determining Region; SIM: Sulphur Indole Motility; SPSS: Statistical Package for Social Science; TSI: Triple Sugar Iron; VP: Voges Proskauer; WHO: World Health Organization; XLD: Xylose Lysine Deoxycholate.
